# Formation and Characterization of β-Lactoglobulin and Gum Arabic Complexes: The Role of pH

**DOI:** 10.3390/molecules25173871

**Published:** 2020-08-25

**Authors:** Ziyuan Wang, Jie Liu, Jian Gao, Mengna Cao, Gerui Ren, Hunjun Xie, Mingfei Yao

**Affiliations:** 1 Beijing Advanced Innovation Center for Food Nutrition and Human Health, Beijing Technology & Business University, Beijing 100048, China; wangziyuan@btbu.edu.cn (Z.W.); liu_jie@btbu.edu.cn (J.L.); 2 School of Food Science and Biotechnology, Zhejiang Gongshang University, Hangzhou 310018, China; 18020080107@pop.zjgsu.edu.cn (J.G.); 18020080045@pop.zjgsu.edu.cn (M.C.); rengerui@mail.zjgsu.edu.cn (G.R.); 3 State Key Laboratory for Diagnosis and Treatment of Infectious Diseases, Collaborative Innovation Center for Diagnosis and Treatment of Infectious Diseases, National Clinical Research Center for Infectious Diseases, The First Affiliated Hospital, College of Medicine, Zhejiang University, Hangzhou 310003, China

**Keywords:** protein–polysaccharide complexes, β-lactoglobulin, gum arabic, turbidity, electrostatic interactions

## Abstract

Protein–polysaccharide complexes have received increasing attention as delivery systems to improve the stability and bioavailability of multiple bioactive compounds. However, deep and comprehensive understanding of the interactions between proteins and polysaccharides is still required for enhancing their loading efficiency and facilitating targeted delivery. In this study, we fabricated a type of protein–polysaccharide complexes using food-grade materials of β-lactoglobulin (β-Lg) and gum arabic (GA). The formation and characteristics of β-Lg–GA complexes were investigated by determining the influence of pH and other factors on their turbidity, zeta-potential, particle size and rheology. Results demonstrated that the β-Lg and GA suspension experienced four regimes including co-soluble polymers, soluble complexes, insoluble complexes and co-soluble polymers when the pH ranged from 1.2 to 7 and that β-Lg–GA complexes formed in large quantities at pH 4.2. An increased ratio of β-Lg in the mixtures was found to promote the formation of β-Lg and GA complexes, and the optimal β-Lg/GA ratio was found to be 2:1. The electrostatic interactions between the NH_3_^+^ group in β-Lg and the COO^−^ group in GA were confirmed to be the main driving forces for the formation of β-Lg/GA complexes. The formed structure also resulted in enhanced thermal stability and viscosity. These findings provide critical implications for the application of β-lactoglobulin and gum arabic complexes in food research and industry.

## 1. Introduction

Protein−polysaccharide complexes have received increasing attention since they possesses multiple advantages over other delivery systems [1]. First, the preparation process is relatively simple, usually originating from electrostatic interactions between two polymers with opposite charges. These formed soluble complexes may further aggregate and precipitate in large quantities [2]. Furthermore, proteins and polysaccharides are mostly food-grade and are easy to obtain. If controlled well, they will exert unique functions and their applications can be very broad in the food industry [3], such as delivery systems for targeted delivering and controlling the release of bioactive compounds [4,5], interface stabilizers and surfactants for emulsions [6,7], as well as surface rheology modulators [8].

Although protein and polysaccharide complexes have been extensively studied previously [1,9,10,11,12,13,14], the interactions between these two materials still require a more systematic study. In this study, protein−polysaccharide complexes were fabricated with β-lactoglobulin (β-Lg) and gum arabic (GA). The β-Lg and GA complexes represent a typical type of protein−polysaccharide complexes. The kinetics of formation and functional properties [15], pH of phase boundaries [13], thermodynamics [16] and structural mechanisms have been characterized previously, but a comprehensive study is still required in order to obtain a complete understanding of their properties. β-Lg is the major protein in cattle and other ruminant milk, with a molecular weight of 18.3 kDa and an isoelectric point of about 5.1. β-Lg has a β-barrel structure consisting of eight antiparallel β-sheets, also known as the calyx structure [17,18]. The calyx structure of β-Lg can automatically bind to hydrophobic molecules and ensure the stability of hydrophobic ligands. β-Lg is a very typical globular protein that is used to study interactions with different polysaccharides. It has been reported that β-Lg and low methoxyl pectin at a ratio of 4:1 can produce complexes at pH 4.5 [19]. The stronger the charge density of the polysaccharide, the higher the degree of complexes that will be formed [19,20,21]. GA is derived from the trunk exudate of the acacia tree of the leguminous family and is also called acacia gum. It is a polysaccharide composed of six carbohydrate moieties and a protein fraction. It also has a “wattle blossom” structure in which a number of polysaccharide units are linked to a common polypeptide chain [13]. As a weak polyelectrolyte carrying carboxyl groups, GA usually shows a negative charge when the pH is above 2.2. Although it does not provide energy, it is a dietary fiber with good water solubility. Moreover, GA has been confirmed to have the function of lowering blood cholesterol [1,22,23]. GA is widely used in the manufacture of various oil-in-water emulsion systems because it has low viscosity and is stable, adhesive and indigestible in the gastrointestinal tract [11,14,24,25].

In this study, the influence of different factors, including protein–polysaccharide ratio, ionic strength, heat and especially the pH, on the formation and properties of β-Lg–GA complexes was investigated using UV–visible (UV–vis) spectroscopy, fluorescence spectroscopy, zeta-sizer, dynamic light scattering, infrared spectroscopy and rotational rheometry. The results will provide important implications for understanding the interaction between proteins and polysaccharides, facilitating the rational design of protein–polysaccharide complexes and evaluating the possibility of further applications of β-Lg–GA complexes in the food industry.

## 2. Materials and Methods

### 2.1. Materials

β-Lactoglobulin (90% β-Lg) was purchased from Sigma. Gum arabic (GA) was purchased from Aladdin Reagent (A108975, Shanghai, China)). HCl (36–38%) was purchased from Shuanglin Chemical Reagent Factory (Hangzhou, China). All other reagents were purchased from Aladdin Reagent and were of analytical grade.

### 2.2. Preparation of β-Lg−GA Mixed Solution and β-Lg–GA Complexes

β-Lg (1 g) and GA (1 g) were dissolved in 100 mL deionized water (*w*:*v*) separately, and the pH of the solutions was adjusted to 7.0 using HCl and NaOH solutions with gradient concentrations, including 1 M, 0.1 M and 0.01 M. They were stored at 4 °C overnight until the polymers were fully dissolved. Then, β-Lg and GA were mixed at different ratios to make the β-Lg−GA mixture. For preparing β-Lg–GA coacervations, β-Lg solution and GA solution were mixed at the ratio of 2:1 and the mixed solution was adjusted by HCl and NaOH solutions until the pH was 4.0. Usually, several drops are needed so that the overall ionic strength will not be influenced. The mixture solution was centrifuged at 17.5 G (TGL-16M, Xiangyi Lab Instruments, Hunan, China) for 15 min and the lower phase was collected and subjected to freeze-drying (SCIENTZ-10N, Xinzhi Biotechnology Inc., Ningbo, China).

### 2.3. Turbidity Measurements

The turbidity of β-Lg−GA mixed solutions was determined by a UV–Vis spectrophotometer (UV-2500, Shimadzu, Tyoto, Japan) at the wavelength of 600 nm (OD 600 nm) under the conditions of different pH values (1.6–7.0), NaCl concentrations (0–50 mmol/L), β-Lg/GA ratios (*w*:*w*, from 1:3 to 3:1) and temperatures (30–50 °C). Turbidity was calculated according to the following Equation (1):

T = −ln (I/I_0_), (1)

where T represents turbidity. I/I_0_ is the ratio of the intensity of the emergent and incident light. Measurements were carried out in triplicate.

### 2.4. Effect of pH on the Structure of β-Lg−GA Complexes

The effect of pH on β-Lg−GA complexes with a β-Lg/GA ratio of 2:1 (0.05%:0.025%) was evaluated using UV–vis spectroscopy under a scanning wavelength ranging from 220 nm to 400 nm.

The effect of pH on β-Lg−GA complexes with a β-Lg/GA ratio of 2:1 (0.05%:0.025%) was also determined by fluorescence spectroscopy (RF-5301PC, Shimadzu, Japan) with the parameters set as follows: (a) endogenous fluorescence: the excitation wavelength was 290 nm and the range of the scanning wavelength was 220–400 nm and (b) synchronous fluorescence: the excitation wavelength was 220 nm. The differences between the excitation wavelength and the initial scanning wavelength of 15 nm and 60 nm were selected.

### 2.5. Effect of pH on the Particle Size and Zeta-Potential of β-Lg−GA Complexes

The zeta potentials of β-Lg solution, GA solution and β-Lg−GA complex solution at different pH were measured by a zeta-sizer (Zeta-sizer Nano-ZS, Malvern, UK) at 25 °C. Using the same instrument, particle size distributions of β-Lg−GA complexes at different pH values were also evaluated. All samples were tested at least for three times in parallel and their mean value was calculated.

### 2.6. Effect of pH on the Viscosity of β-Lg−GA Complexes

The effect of pH on the apparent viscosity of GA solution and β-Lg−GA solution was determined by a rotary rheometer (HAAKEMARS III Thermo Fisher Scientific, Madison, WI, USA). Two plates with the diameter of 60 mm were chosen and the set gap between them was 1.0 mm. Samples were placed between the two plates and equilibrated for two minutes before the apparent viscosity was measured at shear rates of 0.1–100 s^−1^.

### 2.7. Differential Scanning Calorimetry (DSC) and Fourier Transform Infrared (FTIR) Spectroscopy

β-Lg, GA and β-Lg−GA complex powders (2.5 mg) were weighed and used for analysis by DSC (Evo 131, Setaram Instrumentation, France) with an incremental temperature increase of 10 °C/min within the range from 20 °C to 200 °C. An appropriate amount of β-Lg, GA or β-Lg−GA complex solid powders was taken, mixed and grinded with potassium bromide (1/100, *w*/*w*) and made into pellets. Then, they were compressed to form transparent slices in the mold. Each sample was scanned for 32 times by an FTIR spectrometer (Nicolet iS5, Thermo Fisher Scientific) at a resolution of 4 cm^−1^ in the wavelength range of 4000–400 cm^−1^ at room temperature.

## 3. Results and discussion

### 3.1. Effect of pH on Turbidity of β-Lg−GA Complexes

**Figure 1:**
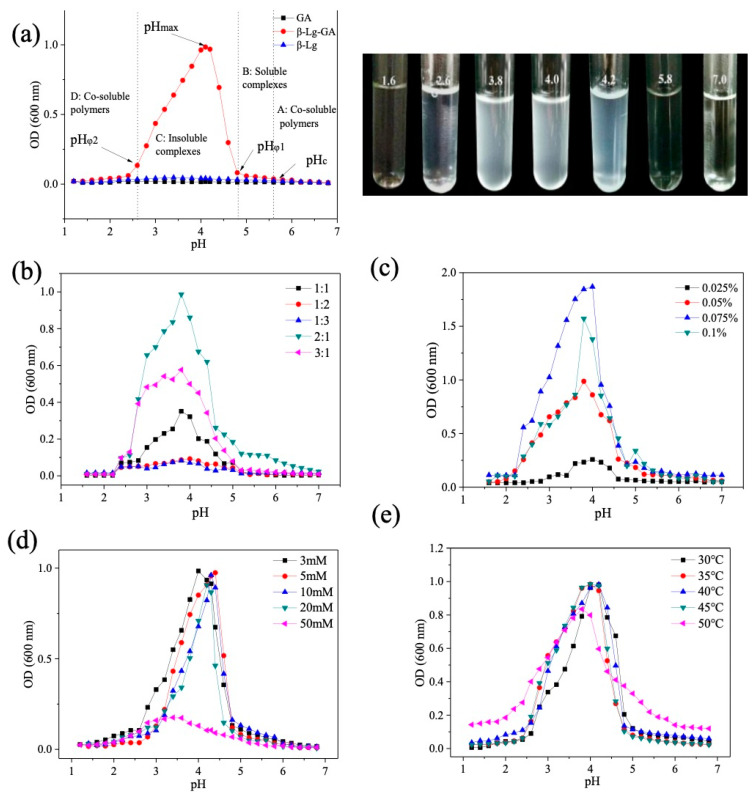
Influence of pH on the turbidity of the mixed solution. (**a**) The turbidity of β-lactoglobulin (β-Lg), gum arabic (GA) and β-Lg−GA mixed solution at different pH values. The concentration of β-Lg and GA solution was 0.025% (*wt*%) and the concentration of the β-Lg−GA mixture solution was 0.05% (wt%), where the ratio of β-Lg and GA was 2:1. (**b**) Effect of concentration on the turbidity of the β-Lg−GA mixed solution at different pH values. The concentration of the β-Lg−GA mixed solution was 0.05% (*wt*%) and the ratios of β-Lg and GA were 1:3, 1:2, 1:1, 2:1 and 3:1, measured at a temperature of 25 °C. (**c**) The influence of concentration of the β-Lg and GA mixture on turbidity at different pH values. The ratio of β-Lg and GA was 2:1 (*w*:*w*) and the concentrations of β-Lg were 0.025%, 0.05%, 0.075% and 0.1%. Turbidity was measured at a temperature of 25 °C. (**d**) Influence of temperature on the turbidity of the β-Lg−GA mixture solution at different pH values. The concentration of the β-Lg−GA mixed solution was 0.05% (*wt*%) and the ratio was 2:1. Turbidity was measured at temperatures of 30, 35, 40, 45 and 50 °C. (**e**) Influence of ionic strength on the turbidity of β-Lg and GA mixed solution at different pH values. The concentration of the β-Lg and GA mixture was 0.05% (*w*%) and the ratio was 2:1. The concentrations of sodium chloride in the solutions were 3, 5, 10, 20 and 50 mM.

Since both β-Lg and GA are weak biological electrolytes, pH can be the main factor contributing to the formation of β-Lg−GA complexes. Therefore, the effect of pH on the turbidity of β-Lg−GA complexes was determined. As shown in Figure 1a, pH did not affect the turbidity of the single β-Lg solution and the single GA solution, although the β-Lg solution had a slight change at pH between 4 and 5, which may be caused by self-association of the protein. Interestingly, the turbidity of β-Lg–GA mixed solution changed dramatically with pH. It can be divided into four regimes with pH decreasing from 7.0 to 0, including co-soluble polymers (A), soluble complexes (B), insoluble complexes (C) and co-soluble biopolymers (D). There were four critical points of pH values. pH_c_ was about 5.6, which was the starting point for the formation of β-Lg−GA complexes. pH_φ_1 was about 4.8, which indicated the sign of forming insoluble complexes. pH_φ_2 was about 2.6, which represented complete dissociation of insoluble complexes. Maximum turbidity was present at pH 4.2, which was identified as pH_max_. Theoretically, the β-Lg−GA complexes cannot be formed at pH_c_ 5.6, since the isoelectric point of β-Lg is 5.2 and it carries a negative charge at pH 5.6, while GA also carries a negative charge, so they will repulse each other. The formation of complexes may be ascribed to the local electrostatic potential interactions between the charged groups of β-Lg and GA. When the pH is below 5.2, both β-Lg and GA carry opposite charges. Electrostatic interactions induced the formation of β-Lg–GA complexes, which will be further investigated in the following section. The increase in turbidity was caused by the formation of β-Lg−GA complexes in large-scale [26]. When the pH was about 4.2, the turbidity reached its maximum value of about 0.997 ± 0.005 and the insoluble complexes dissociated completely at pH 2.6.

Next, we also determined the effect of β-Lg/GA ratio, concentration of β-Lg/GA, temperature and ions on the turbidity of β-Lg–GA complexes in order to understand the system comprehensively. Results are shown in Figure 1b–e. When the ratio of β-Lg/GA was below 1:1, the turbidity was very low during pH change. With the increasing ratio of β-Lg, the turbidity significantly improved at the pH range of 2.6–4.8 (Figure 1b). The turbidity reached the maximum when the ratio of β-Lg/GA was 2:1, likely due to the saturated adsorption of protein on the polysaccharide chain. However, an excessive amount of β-Lg will also decrease the turbidity. It was obvious that the turbidity enhanced with increasing concentrations of β-Lg−GA complexes in the solution and pH_max_ showed a slight shift to the right (Figure 1c). When the concentration increases, a lot of anti-balance ions will be released into the solution, which may shield the potential points on the surface of biomacromolecules and inhibit the aggregation of the complexes [18]. High NaCl concentrations will destroy the system according to Figure 1d. When the ion concentration was 50 mM, the turbidity was significantly reduced. NaCl will be ionized to provide Na^+^ and Cl^−^ in the aqueous solution, and there is a competitive relationship between Na^+^ and the positive charge groups of β-Lg molecules to adsorb to the side chain of GA. Besides, NaCl also reduces the intramolecular charge repulsion between the chains of gum arabic, thereby reducing the gel strength of gum arabic. The β-Lg−GA complexes also exhibited high stability at high temperatures (Figure 1e), which will be further characterized by DSC as described in the following section.

### 3.2. Effect of pH on the Structures of β-Lg−GA Mixed Solution

**Figure 2:**
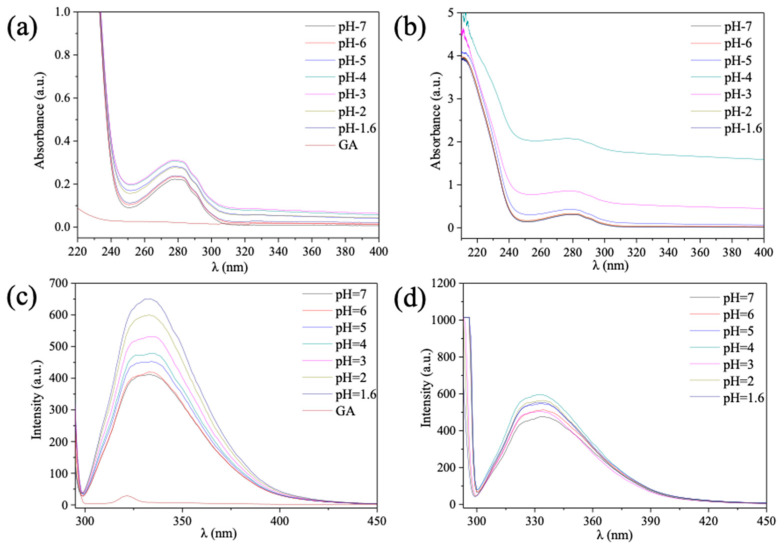
(**a**) UV–Vis spectroscopy of β-Lg (0.025%) at different pH and UV-Vis spectroscopy curve of GA (0.025%), (**b**) UV–Vis spectroscopy of β-Lg−GA composite (0.05%, 2:1) at different pH values, (**c**) fluorescence emission spectra of β-Lg (0.025%) at different pH values and spectra of GA (0.025%) and (**d**) fluorescence emission spectra of β-Lg−GA mixed solution (0.05%, 2:1) at different pH values.

The structures of β-Lg, GA and β-Lg−GA complexes were evaluated by using UV–vis spectroscopy. Figure 2 a showed that there was no UV absorbance observed for GA in the wavelength range of 220–400 nm (Figure 2). For β-Lg, the curve of UV absorbance did not change under different pH conditions. pH significantly enhanced the UV absorbance of β-Lg−GA complexes at pH 3.0 and pH 4.0, although the shape of curve did not change (Figure 2b). This result was consistent with the high turbidity of the β-Lg−GA mixed solution which appeared within the range of pH 3.0–4.0, when β-Lg−GA complexes formed in large quantities.

When proteins interact with other molecules in the solution, the fluorescent intensity of the solution system tends to change. Normally, to explore the conformation of proteins and their interactions with molecules, the fluorescence of tryptophan residues of proteins would be detected [27]. In this study, in order to avoid the influence of other amino acid residues, 290 nm was chosen as the excitation wavelength. The fluorescence intensity of GA seemed to be undetectable and had only a tiny peak around 320 nm. The peak of β-Lg UV absorbance appeared at 330 nm. The maximum fluorescence intensity reduced when the pH of the β-Lg solution increased (Figure 2c). For β-Lg−GA complexes, a spectroscopic redshift was observed, indicating that the interactions between GA and β-Lg altered the conformation of β-Lg, pushing the tryptophan residue to a more hydrophobic environment. Moreover, it was again identified that the maximum fluorescent intensity was present at the pH value of 4.2.

### 3.3. Effect of pH on the Zeta-Potential and Size Distribution of β-Lg−GA Complexes

Zeta-potential is frequently used to understand polysaccharide and protein complexation [28]. In order to illustrate the mechanisms of the maximal amount of β-Lg−GA complexes forming at pH 4.2, we further investigated the change of zeta-potential and size distribution at different pH conditions.

**Figure 3:**
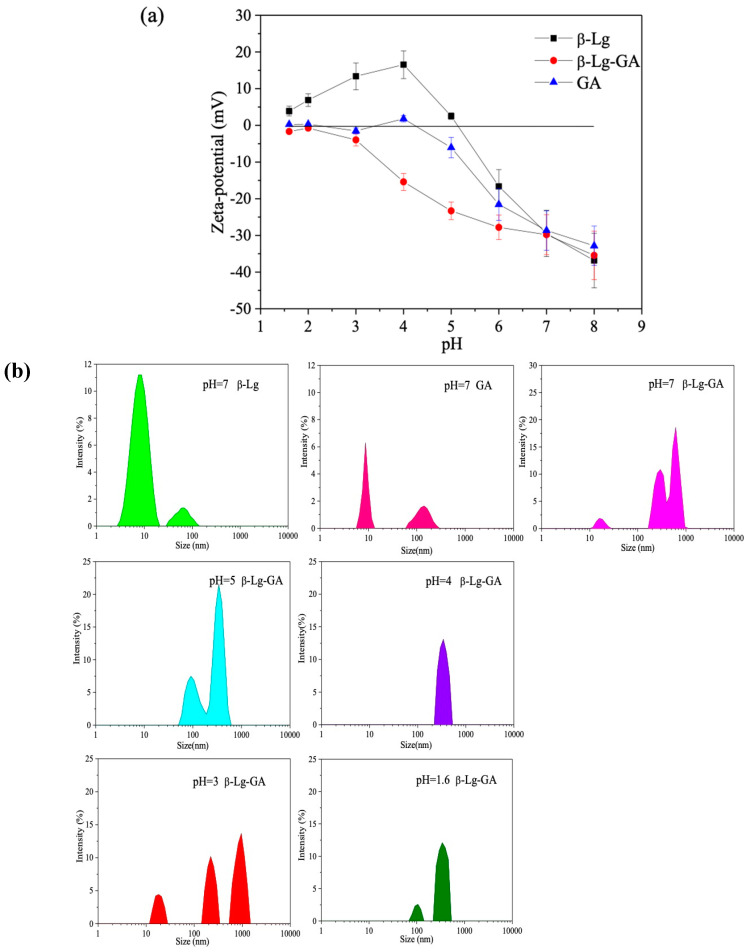
Zeta-potential (**a**) and particle size (**b**) of β-Lg (0.025%), GA (0.025%) and β-Lg–GA solution (0.05%, 2:1) varies at different pH values. Each sample was measured in triplicate. Error bars indicate the standard deviation of three different measurements for each sample.

Figure 3a shows the curves of zeta-potential of β-Lg, GA and β-Lg−GA at different pH values. GA carries negative charge due to the presence of a carboxyl group, and its zeta-potential varied from −1.67 mV to −35.43 mV when the pH increased from 1.6 to 8.0. The β-Lg solution maintained a positive charge at low pH, while it became negatively charged when the pH was above 5.2. At pH 4.2, β-Lg had its highest positive zeta-potential so that a great quantity of β-Lg−GA complexes can be formed with GA, since the electrostatic attractions between opposite charges can promote the formation of β-Lg−GA complexes. The β-Lg−GA complex solution carried a negative charge, indicating that there was still a number of free carboxyl groups that did not interact with β-Lg.

Figure 3b showed size distributions of β-Lg and GA at pH 7.0 and the size distribution of β-Lg −GA complexes at different pH values. Two separate regions were observed in single β-Lg solution and single GA solution. Bovine β-Lg is a small protein, consisting of 162 amino acids with a monomeric mass of ~18,300 Da. One monomer of bovine β-Lg contains eight β-strands that form the central antiparallel β-sheet calyx and a ninth β-strand that is involved in dimer formation. Each monomer contains five cysteine residues and four of them form two disulfide bridges to fold β-Lg [29]. A molecule of free β-Lg had a hydrodiameter of about 4.86 nm and the diameter reduced to 2 nm in solution at medium pH at room temperature. Therefore, the region with smaller particle size was recognized as β-Lg or GA molecule [30]. The region with larger size was present due to the self-association of the protein in the solution, whereas the β-Lg–GA solution showed three regions at 15.7 nm, 295 nm and 615 nm separately. According to the results of the turbidity experiments, it could be a mixture of β-Lg, GA, β-Lg–GA complexes and an aggregation of the polysaccharide. When the pH was 5, the region with the smallest size disappeared and two separate regions of sizes 92 nm and 342 nm were left. Interestingly, only one region was left and the average size was 395 nm when the pH was 4, indicating that all β-Lg and GA contributed to the formation of β-Lg–GA complexes. When the pH was reduced to 3 or 1.6, particles were separated into several regions.

### 3.4. Effect of pH on the Rheological Properties of β-Lg−GA Complexes

**Figure 4:**
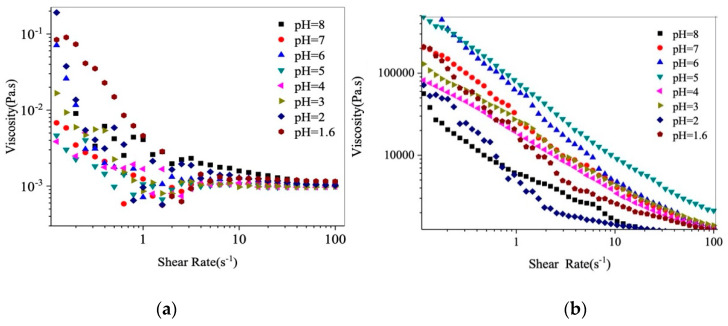
Effect of pH on the viscosity of GA (**a**) and β-Lg−GA complexes (0.5%, 2:1) (**b**).

In this section, the rheological properties of β-Lg−GA complexes are examined. Figure 4a shows the change in the apparent viscosity of the GA solution. The apparent viscosity decreased when the shear rate increased. GA exhibited higher viscosity at lower pH when the shear rate was below 1 s^−1^. The apparent viscosity of β-Lg−GA solution also decreased with increases in the shear rate as shown in Figure 4b, indicating that it is a non-Newtonian fluid [31]. When the pH was in the range of 1.6–4, the viscosity of β-Lg−GA complexes increased as the pH increased. When the pH was in the range of 5–8, the viscosity of the complexes decreased as the pH increased. At the same shear rates, the highest viscosity was generated at pH 6.0 and the lowest viscosity was present at pH 2.0.

The viscosity of the β-Lg−GA complex system was around 10^6^-fold higher than that of the GA solution. This was because the electrostatic interactions between β-Lg and GA are strong while the surface charge of the particle is close to neutral. Therefore, the particles tend to aggregate so that the apparent viscosity is very high. At higher shear rates, the physical forces maintaining the structures of complexes are destroyed, leading to the decrease in viscosity [2,32,33,34].

### 3.5. Thermal Behavior and Fourier Transform Infrared Spectroscopy

**Figure 5:**
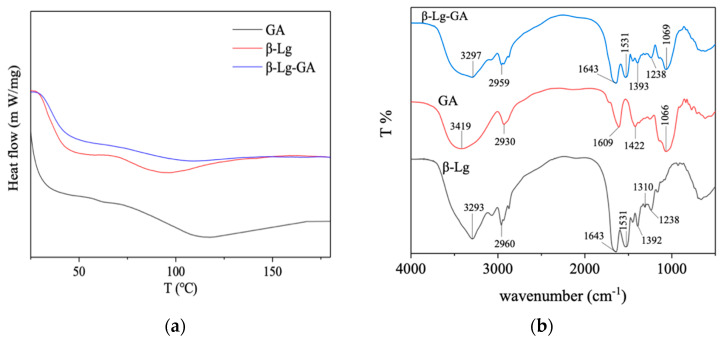
(**a**) Differential scanning calorimetry (DSC) thermograms of β-Lg, GA and β-Lg−GA complexes. (**b**) FT-IR spectra of β-Lg, GA and β-Lg−GA complexes.

The thermal behavior and FTIR spectrum of the β-Lg−GA complexes were determined by calorimetric (DSC) and spectroscopic (circular dichroism, FTIR) methods. The main characteristic thermal transitions include a glass transition for the amorphous fraction, a melting transition for the crystalline fraction and a transition due to crystallization [35]. The DSC thermograms of β-Lg, GA and β-Lg−GA complexes are shown in Figure 5a. No melting peaks were found in any of these curves, indicating that they were without a crystalline structure. The DSC thermogram of GA exhibited an endothermic peak at 123 °C, which accounted for its glass transition temperature. The thermogram of β-Lg showed a peak at 95 °C and β-Lg−GA showed a broad peak at 116 °C, which may be associated with the protein denaturation transitions. The protein transition temperature of materials increased after β-Lg−GA formed. DSC has been considered to be a very useful tool for studying the thermal properties of proteins and protein denaturation process [36,37]. When a protein is subjected to programmed heat, the endothermic peaks that appear reveal the denaturation transitions. The enhanced heat stability may be due to the rearrangement of the conformation of the protein and the formation of a stable network structure with GA. The globular structure of β-Lg–GA also reinforces the stability [9,38].

The FTIR spectrum of β-Lg, GA and β-Lg–GA complexes are shown in Figure 5b. GA is a heteropolysaccharide of arabinogalactan and glycoproteins [35]. The broad band at 3419 cm^−1^ was an indicator of amino groups. The peak at 2930 cm^−1^ represented free carboxyl groups with a negative charge. The absorption bands at 1609 cm^−1^ and 1422 cm^−1^ represented asymmetric and symmetric stretching vibration of carboxyl groups. The absorption band at 1066 cm^−1^ represented stretching vibrations of carbonyl groups [39].

The main signals in the IR spectrum of β-lactoglobulin may be attributed to the protein backbone. As a globular protein, the secondary amines (R_2_NH) of β-Lg showed a weak absorption band at 3293 cm^−1^. The absorption band at 1643 cm^−1^ was believed to be from C=O stretching vibration with minor contributions from the out-of-phase CN stretching vibration, CCN deformation and NH in-plane bend. The region from 1238 to 1393 cm^−1^ was mainly contributed by the NH bending on the side chain structure of β-Lg [40].

The band of carboxyl groups in GA and the amino group in β-Lg shifted after the formation of β-Lg–GA complexes (carboxyl peak from 2930 cm^−1^ to 2959 cm^−1^; amino peak from 3293 cm^−1^ to 3297 cm^−1^), which may indicate the electrostatic interactions between amino groups of β-Lg as a positive group and free carboxylic groups of GA as a negative group. Interestingly, the absorption bands at 1313 cm^−1^ (in β-Lg), 1066 cm^−1^ (in GA) and 1423 cm^−1^ (in GA), which were present in single β-Lg or single GA, disappeared in the infrared spectrum of the β-Lg−GA complexes, which also indicated that the electrostatic interactions between the carboxylic group in GA and NH^3+^ group on the side chain in β-Lg were the main forces leading to the formation of the β-Lg−GA complexes [33].

## 4. Conclusions

In conclusion, pH played a critical role in the formation of the β-Lg/GA complexes and 4.2 was considered to be the most favorable pH condition. At pH 4.2, β-Lg exhibited its strongest positive zeta-potential so that a large quantity of β-Lg−GA complexes can be formed with GA. Besides, 2:1 was believed to be the optimal ratio of β-Lg/GA, at which the adsorption of the protein on the polysaccharide chain was saturated. Although high NaCl concentrations (≥50 mM) will destroy the system, the β-Lg−GA complexes had good stability at high temperatures, which may be associated with the formation of firm networks between β-Lg and GA. The electrostatic interactions between the COO^−^ group in GA and NH_3_^+^ in β-Lg was confirmed to be the main force for the formation of the β-Lg−GA complexes. This assembled protein–polysaccharide complexes will be applied for the delivery of multiple bioactive compounds in future studies.
